# Soluble Receptor of Advanced Glycation End Products and Cardiometabolic Markers in Children

**DOI:** 10.7759/cureus.81680

**Published:** 2025-04-03

**Authors:** Celia Aradillas-García, Mariela Vega-Cárdenas, Marisol Vidal-Batres, Diana P Portales-Pérez, Armando Gomez-Ojeda, Claudia Luevano-Contreras

**Affiliations:** 1 Faculty of Medicine, Universidad Autónoma de San Luis Potosí, San Luis Potosí, MEX; 2 Coordination for the Innovation and Application of Science and Technology, Universidad Autónoma de San Luis Potosí, San Luis Potosí, MEX; 3 Faculty of Chemical Sciences, Universidad Autónoma de San Luis Potosí, San Luis Potosí, MEX; 4 Department of Medical Sciences, University of Guanajuato, León, MEX

**Keywords:** body mass index, cardiometabolic, childhood obesity, obesity biomarkers, receptor for advanced glycation end products

## Abstract

Background

The soluble receptor for advanced glycation end products (sRAGE) is a proposed obesity biomarker, but its role in childhood has not been fully elucidated. This study aimed to evaluate the association between sRAGE levels and cardiometabolic factors in children according to body mass index (BMI).

Introduction

This cross-sectional study included 124 children aged 6-9 years, categorized as normal weight (n=72) and overweight or with obesity (n=52). Anthropometric and clinical measurements included weight, height, BMI, waist circumference (WC), neck circumference, and systolic and diastolic blood pressure (SBP and DBP). Fasting blood samples were collected to measure glucose, lipid profile, uric acid (UA), and sRAGE levels using standardized methods. We also calculated the triglyceride-glucose index (TyG), visceral adiposity index (VAI), and triglyceride-to-HDL-C (TG:HDL-C) ratio.

Results

Children who were overweight and obese exhibited lower sRAGE levels compared to the normal-weight group (p= 0.02) and higher WC, NC, UA, HDL-C, TG, SBP, DBP, TyG, TG:HDL-C ratio, and VAI than the normal-weight group (p<0.01). No differences in glucose, creatinine, and cholesterol were found. Finally, sRAGE levels showed correlations with BMI (r=-0.358, p=0.03), WC (r=-0.242, p=0.00), and HDL-cholesterol (r=0.207, p=0.01).

Conclusions

Children with obesity presented lower sRAGE levels and higher cardiometabolic risk markers, including higher WC, TG, TG: HDL-C ratio, TyG index, VAI score, and lower HDL-C than the normal weight group.

## Introduction

Childhood obesity is a significant public health issue in Mexico and worldwide [1,2]. Trends across different countries are heterogeneous, with obesity rates stabilizing in high-income countries while accelerating in more disadvantaged areas [3]. Obesity in children is influenced by genetic, environmental, and behavioral factors and is closely associated with metabolic disturbances [4].

Cardiometabolic risk (CMR) includes factors such as high blood pressure, insulin resistance (IR), hyperinsulinemia, dyslipidemia, and type 2 diabetes [5,6]. Children with obesity are more likely to remain obese into adolescence and adulthood, with up to a fivefold increased risk compared to those with normal weight [7]. This is of even greater concern in patients with additional risk factors. The question arises as to whether the early detection of obesity in the pediatric population will reduce future CMR in adulthood.

Several indices have been proposed to improve early detection of CMR. The triglyceride-glucose (TyG) index has been identified as a reliable marker of IR [8]. Earlier IR detection using the TyG index could help identify children at risk of type 2 diabetes. The visceral adiposity index (VAI), on the other hand, is linked to a higher risk of cardiovascular disease (CVD) and can predict metabolic syndrome (MetS) in children and young adults [9,10]. Furthermore, the VAI can be used as a marker of visceral adiposity and CMR in pediatric patients. In addition, the triglyceride-to-high-density lipoprotein cholesterol (TG:HDL-C) ratio has been reported as an accurate measure for MetS prediction [11]. The above indices are characterized by higher sensitivity and specificity than conventional anthropometric parameters and could significantly improve the assessment of CMR associated with childhood obesity.

Fat mass plays an essential role in inflammatory cascades; therefore, inflammation is now considered one of the physiological mechanisms related to obesity as well as its comorbidities [12]. Inflammation and oxidative stress are related to advanced glycation end products (AGEs), a heterogeneous group of compounds produced by non-enzymatic reactions between an amino acid and a reduced sugar when proteins or lipids are glycated after exposure to sugars. Chronic diseases, such as CVD, type 2 diabetes, metabolic syndrome, chronic kidney disease, and neurodegenerative diseases, strongly correlate with AGEs [13]. AGEs can be produced endogenously through hyperglycemia and oxidative stress or acquired exogenously by ingesting foods high in AGEs [14]. These compounds activate a transmembrane receptor called Receptor for Advanced Glycation End Products (RAGE), leading to increased expression of cytokines, adhesion molecules, and reactive oxygen species (ROS). Consequently, pro-inflammatory pathways, procoagulant responses, and oxidative stress are triggered [15].

A soluble isoform of RAGE, known as soluble RAGE (sRAGE), has been identified in the plasma. sRAGE functions as a competitive decoy molecule for AGEs, preventing the overactivation of RAGE and subsequent inflammation [16]. As a result, sRAGE levels may serve as biomarkers of inflammation and endothelial dysfunction [17]. Additionally, an inverse correlation has been observed between RAGE and sRAGE levels in clinically healthy children without inflammation [18]. RAGE activity in the body is partially mediated by ligand activation [19]. Therefore, the AGE-RAGE axis has been proposed as a mechanism that contributes to metabolic alterations associated with adiposity. Given its involvement in inflammatory processes, it is essential to understand the association between sRAGE and obesity, particularly in the pediatric population.

In adolescents with obesity, a correlation between body mass index (BMI) and sRAGE has been established, indicating a preclinical phase of metabolic alterations that may result in compromised endothelial function [20]. Other pediatric studies have demonstrated that reduced sRAGE levels in obese individuals are associated with a waist-to-height ratio, glucose levels, and adiponectin levels, implying that a decrease in sRAGE may precede the onset of metabolic syndrome [21]. Obesity and an elevated homeostatic model assessment of insulin resistance index are inversely related to sRAGE levels in adolescents [22]. Moreover, the relationship between sRAGE and CMR markers, such as BMI, waist circumference, blood pressure, triglycerides (TG), fasting glucose, and insulin resistance index, has been documented in children with obesity [23]. An increased AGE/sRAGE ratio and decreased sRAGE levels have been observed in children and adolescents with obesity. Notably, women in this group exhibited more cardiovascular risk factors and higher sRAGE levels in relation to TG/HDL-C levels [24]. A recent study reported that low sRAGE levels were associated with an elevated risk of hypertension, excess weight, and a higher waist-to-height ratio in children and adolescents [25].

The aforementioned discussion underscores the urgent need to determine whether sRAGE serves as a biomarker that can facilitate a deeper understanding of the specific pathophysiology and improve the clinical management of cardiometabolic risk associated with obesity in children. The observed correlation between reduced sRAGE levels and adiposity markers in youth indicates that the AGE-RAGE axis may be disrupted early in life, suggesting that sRAGE could be a significant marker for stratifying pediatric cardiometabolic risk.

Given the established correlation between sRAGE levels and obesity markers, investigating this relationship in prepubertal children is crucial. This age group has been underrepresented in research, raising questions about the early impact of obesity on sRAGE levels and CMR. Consequently, this study sought to assess the levels of sRAGE and cardiometabolic markers in children aged 6-9 years, categorized by different BMI classifications, within a cohort of overweight and obese children, in comparison to their lean counterparts.

## Materials and methods

Data collection

The information used in this study came from the large-scale project called “Identification of genetic profiles, proteomics, and risk factors associated with non-communicable diseases and their comorbidities". The subproject was registered as "Prevalence and risk factors associated with non-communicable diseases in the school population of the state of San Luis Potosi" with the Bioethics and Research Committee of the State of San Luis Potosí and received approval in 2015 (SLP/003-2015), prior to data collection.

Participants were selected from public schools in the metropolitan areas of San Luis Potosí, México, who attended school during the 2015-2017 academic years. Eligibility required participants to be residents of San Luis Potosí, Mexico. Children with a documented medical history of diabetes and/or CVDs were not included.

The sample was obtained using stratified random sampling at each education level; two schools were randomly selected from the complete list of schools in the state of San Luis Potosí, classified according to marginalization indexes, as previously described in the study by González-Cortés et al. [26]. In addition, the sample size for this study was calculated using a 97% confidence interval and an acceptable margin of error of 5%, resulting in a final sample size of 124 children aged 6-9 years. Each participant underwent a clinical examination consisting of (a) anthropometric measurements (weight and height), (b) clinical measurements (BP), and (c) blood sample collection (after 12 hours of fasting to determine biochemical parameters). The parents or legal guardians of all participants provided written informed consent in Spanish for the use of the data for research purposes and public availability.

Procedures

All measurements were conducted by trained and standardized health professionals. A registered dietitian assessed anthropometric variables as previously described [26]. The measurements included weight (kg), height (cm), BMI (kg/m^2^), waist circumference (WC) (cm), and neck circumference (NC) (cm).

Electronic scales (TANITA UM-81) and stadiometers (Seca 213) were placed on a flat surface to measure weight and height. Participants wore light clothing, without shoes or socks, and the Frankfurt plane was used to measure height. BMI was calculated using Quetelet’s formula. Z-scores based on age and gender were used to categorize BMI using the World Health Organization (WHO) AnthroPlus program. The WHO classification was applied as follows: obesity (> 2 Z-score), overweight (> 1 Z-score), normal weight (-1 to 0 Z-score), thinness (< -2 Z-score), and severe thinness (< -3 Z-score).

WC was measured using a LUFKIN Executive thin-line (2 m) metal tape at the midpoint between the iliac crest and the ribcage. The measurement was taken at the narrowest part of the abdomen at the end of a normal expiration, with the subject in a standing position. For NC measurement, the LUFKIN Executive thin-line 2 m metal tape was also used, with participants standing upright and facing forward. The measurement was taken just below the thyroid cartilage.

We evaluated systolic blood pressure (SBP) and diastolic blood pressure (DBP) using the HBP-1300 OMRON portable monitor (OMRON, Kyoto, Japan). We used three cuff sizes based on the participant's brachial circumference. The cuff was placed at the midpoint between the olecranon and the acromion, ensuring the subject remained relaxed in a seated position. Measurements were always taken on the right arm, with participants seated for five minutes before recording the first reading [27]. Following these procedures, three measurements were taken at five-minute intervals, and the average of the last two measurements was recorded.

Blood samples (6 mL) were collected using a BD Vacutainer system. Children fasted for 12 hours prior to collection. Blood samples were processed to determine fasting plasma glucose, total cholesterol (TC), high-density lipoprotein cholesterol (HDL-C), low-density lipoprotein cholesterol (LDL-C), triglycerides (TG), uric acid (UA), and creatinine. Analyses were conducted using a BS 300 Mindray® autoanalyzer (Mindray Bio-Medical Electronics, Shenzhen, China), and fasting plasma glucose was measured using the glucose peroxidase method. All analyses were performed in a certified clinical laboratory. sRAGE levels were determined by ELISA.

The CMR indexes were calculated according to the following formulas:

TG:HDL-C ratio = TG (mmol/L)/ HDL-C (mmol/L) [11]

TyG index = Ln [(TG * glucose) / 2] (TG and HDL-C in mg/dL) [8]

VAI calculations were performed using the following formulas:

Male: VAI = ((WC / 39.86) + (1.88 * BMI)) * (TG / 1.03) * (1.31 / HDL-C)

Female: VAI = ((WC / 39.58) + (1.89 * BMI)) * (TG / 0.81) * (1.52 / HDL-C) 

TG and HDLC are measured in mmol/L, WC in centimeters, and BMI in kg/m^2^ [9,11]. 

Statistical analysis

Continuous variables were expressed as mean ± standard deviation. Comparisons between normal weight and overweight/obesity groups were made using Student’s t-test. Spearman correlation coefficients assessed relationships between sRAGE and cardiometabolic markers. Significance was set at p<0.05. All statistical analyses were performed with GraphPad Prism version 8.0.2 (GraphPad Software, San Diego, CA).

## Results

In accordance with BMI, the total sample of 124 children (51.6% boys and 48.4% girls) was divided into two groups: 72 individuals with normal weight and 52 with overweight or obesity. The participants' ages ranged from 6 to 9 years, with a mean age of 7.9 ± 0.9 years. The main anthropometric, clinical, and biochemical parameters of the 124 individuals assessed in this study are listed in Table 1.

**Table 1:** General characteristics of the study population. Waist c., waist circumference; Neck c., neck circumference; SBP, systolic blood pressure; DBP, diastolic blood pressure; sRAGE, soluble receptor for advanced glycation end products; HDL-C, high-density lipoprotein cholesterol; LDL-C, low-density lipoprotein cholesterol; TG, triglycerides; TyG, triglyceride and glucose; VAI, visceral adiposity index. Student's t-test was performed.

Variables	Normal weight N=72		Overweight and obesity N=52		Significance		
	Media	SD	Media	SD	t	dif	p-vale
Waist c. (cm)	55.9	5.2	72.0	8.7	12.2	-16.2	0.01
Neck c. (cm)	27.2	1.7	30.1	2.2	8.3	-2.9	<0.001
Glucose (mg/dL)	87.9	5.5	88.7	8.0	0.7	-0.8	0.48
Creatinine (mg/dL)	0.6	0.1	0.5	0.1	1.0	-0.0	0.29
Uric Acid (mg/dL)	4.1	0.8	4.9	1.1	5.1	-0.8	<0.001
Total Cholesterol (mg/dL)	148.5	24.8	156.2	24.8	1.7	-7.7	0.09
HDL-C (mg/dL)	56.8	10.2	50.0	9.4	3.7	6.7	0.00
LDL-C (mg/dL)	79.8	21.2	83.6	20.4	1.0	-3.8	0.31
TG (mg/dL)	59.5	14.3	112.6	34.3	7.1	-53.0	<0.001
SBP (mmHg)	103.2	9.2	111	12	4.0	-7.8	<0.001
DBP (mmHg)	59.4	8.3	62.9	9.5	2.1	-3.4	0.03
sRAGE (pg/dL)	2950.9	1038.9	2519	843.1	2.2	385.4	0.02
TG: HDL-C Ratio	1.08	0.3	2.3	0.9	7.2	-1.3	<0.001
TyG index	4.2	0.1	4.5	0.2	7.3	-0.2	<0.001
VAI score	0.5	0.2	1.5	0.8	7.5	-0.9	<0.001

We compared anthropometric, clinical, and biochemical variables among the BMI categories: normal weight, overweight, and obesity. As expected, the anthropometric variables WC and NC were significantly different across BMI categories. The group with overweight and obesity had higher levels of SBP, TG, UA, TG: HDL-C ratio, TyG index, and VAI score compared to the group with normal weight (p<0.001). In addition, the group with obesity had lower HDL-C and sRAGE levels than the group with normal weight (p<0.001). No significant differences were found in fasting plasma glucose or total cholesterol. 

Results by sex showed that boys had higher fasting plasma glucose levels (90.5± mg/dl) than girls (85.8± mg/dl) (p<0.001), as shown in Table 2.

**Table 2:** Comparison of anthropometric, clinical, and biochemical variables in boys and girls. Waist c., waist circumference; Neck c., neck circumference; SBP, systolic blood pressure; DBP, diastolic blood pressure; sRAGE, soluble receptor for advanced glycation end products; HDL-C, high-density lipoprotein cholesterol; LDL-C, low-density lipoprotein cholesterol. TG, triglycerides; TyG, triglyceride and glucose; VAI, visceral adiposity index.

Variables	Boys N=64		Girls N=60		Significance		
	Media	SD	Media	SD	t	dif	p-vale
BMI (Kg/m^2^)	16.8	3.6	17.7	3.0	0.3	0.2	0.75
Waist c. (cm)	63.0	11.4	62.4	9.7	0.3	0.5	0.77
Neck c. (cm)	28.7	2.5	28.2	2.4	1.0	0.4	0.29
Glucose (mg/dL)	90.5	6.3	85.8	6.3	4.1	4.6	<0.001
Creatinine (mg/dL)	0.6	0.1	0.6	0.1	1.8	0.0	0.06
Uric Acid (mg/dL)	4.4	1.1	4.5	1.0	0.3	-0.4	0.69
Total Cholesterol (mg/dL)	152.9	24.6	150.5	25.6	0.5	2.4	0.59
HDL-C (mg/dL)	55.5	10.9	52.3	9.7	1.7	3.1	0.09
LDL-C (mg/dL)	82.6	21.4	80.2	20.4	1.9	2.5	0.51
TG (mg/dL)	74.1	41.9	90.0	54.0	1.8	-15.9	0.06
SBP (mmHg)	106.8	10.6	106.1	11.8	0.3	-0.7	0.71
DBP (mmHg)	59.1	9.7	62.8	7.6	2.3	-3.7	0.01
sRAGE (pg/dL)	2675.1	936.2	2911.1	1012.1	1.3	-236.0	0.17
TG: HDL-C Ratio	1.4	1.1	1.8	1.1	1.6	-0.3	0.09
TyG index	4.3	0.2	4.4	0.2	1.4	-0.0	0.15
VAI score	0.7	0.6	1.2	0.6	4.0	-0.5	<0.001

However, the mean fasting plasma glucose values for both boys and girls were within normal ranges. Overall, girls exhibited a worse metabolic profile, with a higher TyG index, VAI score and TG: HDLC-c ratio than boys.

**Figure 1:**
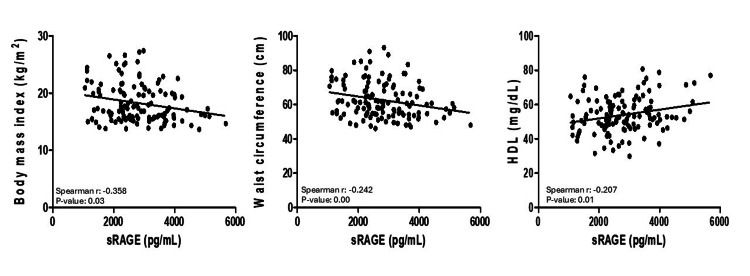
Correlation between soluble RAGE and adiposity markers. sRAGE, soluble receptor for advanced glycation end products; HDL-C, high-density lipoprotein cholesterol. TG, triglycerides; TyG, triglyceride and glucose; VAI, visceral adiposity index. Associations between soluble RAGE and BMI, WC, and VAI were analyzed using Pearson's correlation.

Regarding potential associations between sRAGE and other cardiometabolic factors, sRAGE was found to have a negative correlation with BMI (r =-0.23) (p=0.01) and WC (r = -0.24) (p<0.01), while positive correlations were observed with HDL-C (r = 0.24) (p=0.01) and creatinine (r = 0.18) (p=0.04) as shown in Figure 1.

Additionally, UA, a biochemical marker that could indicate cardiovascular risk, showed a positive correlation with BMI (r =0.48), SBP (r=0.22), TG (r = 0.25), and fasting plasma glucose (r = 0.27) (p<0.01). Finally, no significant correlation was found between sRAGE and CMR indices.

## Discussion

Low-grade inflammation in childhood obesity is characterized by an imbalance in immune cells and release of inflammatory markers. By competing with RAGE for ligand binding, sRAGE prevents inflammatory responses [28]. A high BMI is generally assumed to represent the overall amount of body adipose tissue (AT), and it has been reported that in obesity, there is a higher accumulation of AGEs and increased levels of RAGE in AT than in their lean counterparts [19]. The precise role of RAGE in AT is unknown; the proposed mechanisms are the link between RAGE expression and hypertrophy, and the induction of macrophage activation [15]. In our study, we found that sRAGE levels were higher in the normal weight group than in the obese group, suggesting that the risk may be related to an increase in BMI.

When we stratified the cohort by sex, our results showed no significant changes in sRAGE, which is consistent with the findings of Flores-Ramirez et al. [24], indicating that low levels of sRAGE are associated with obesity. This could be explained by the proposal that higher AGE levels are found due to the increased amount of fat mass. Several authors support the notion that obesity leads to a higher endogenous production of AGEs, and sRAGE acts as a decoy for these molecules, thereby reducing their concentration [21-23]. However, Rodriguez-Mortera reported that adolescents with obesity had higher dietary AGE intake accompanied by lower sRAGE but also lower circulating AGE levels [20]. 

We observed that the groups with overweight and obesity had higher values of UA, TG, SBP, and DBP, as well as lower HDL-C levels than those with normal weight. It is important to mention that our population was metabolically healthy, including the obese group. This suggests that decreased sRAGE levels associated with obesity may serve as an early marker for the onset of metabolic alterations. In accordance with this theory, Gurecká et al. [21] reported that metabolically healthy obese adolescents present significantly lower sRAGE levels in comparison with their lean counterparts, and similarly, with our results, the normal weight groups showed lower levels of the cardiometabolic risk markers. Corica et al. [23] found a strong negative correlation between BMI and sRAGE. Our study also identified a strong negative correlation between these variables, with no other associations observed with biochemical variables. This finding indirectly supports the hypothesis that sRAGE concentration decreases as adiposity increases.

Previous studies have evaluated the significance of expressing the SS genotype of the RAGE gene (rs2070600) polymorphism in adolescents [22]. Additionally, in a previous study conducted by our group, we found higher relative expression of RAGE and lower levels of sRAGE in the obesity group compared to overweight children, which positively correlated with HDL-C levels [29]. Moreover, the present study showed a positive association between sRAGE and HDL-C, which could indicate a preclinical alteration in the lipid profile of this population. We also examined the associations between sRAGE levels and indices of CMR, such as the TG:HDL-C ratio, TyG index, and VAI score. We selected indices based on routine biochemical parameters, such as TG, HDL-C, and glucose, with the addition of anthropometric data. Pediatric research has largely relied on proxy measures of adiposity, such as BMI and WC, rather than on directly measuring VAI, which has been proposed as a predictor of an unhealthy metabolic phenotype in children and adolescents [30]. Although we observed differences between overweight and obese children and those with normal weight, we found no association between sRAGE and VAI. Similarly, no associations were found between sRAGE and the other CMR indices.

This study has some limitations, such as the fact that different isoforms of sRAGE were not measured, and we did not analyze other components of the AGE-RAGE axis. Nevertheless, our study has strengths: the sample size with sufficient statistical power, the health evaluation of children, which implied the assessment of cardiometabolic markers, and the pediatric age of the participants. 

## Conclusions

Our results suggest that obesity is associated with low sRAGE levels in childhood. Our study found that the group with obesity showed higher CMR markers, as shown by a higher WC, TG, and TG:HDL-C ratio, TyG index, and VAI score, and lower HDL-C and sRAGE than the normal weight group. The above highlights that measuring sRAGE levels could provide a biomarker to track the activity of the AGE-RAGE pathway.

In our study, sRAGE levels were weakly associated with WC and HDL-C levels. The consideration that increased sRAGE levels are a sign of metabolic health should be handled with caution, as should the interpretation of isolated values of this receptor in the absence of measurement of AGEs or any other marker of the AGE-RAGE axis. Long-term follow-up studies are needed to prove that sRAGE levels could be a sign of cardiometabolic risk factors in children before they reach puberty. Future research should evaluate the changes in sRAGE levels at different stages of the pediatric age group and after the appearance of RCM factors.
